# Novel adipokine asprosin modulates browning and adipogenesis in white adipose tissue

**DOI:** 10.1530/JOE-20-0503

**Published:** 2021-03-09

**Authors:** Yanli Miao, Haojie Qin, Yi Zhong, Kai Huang, Caijun Rao

**Affiliations:** 1 Department of Cardiology, Union Hospital, Tongji Medical College, Huazhong University of Science and Technology, Wuhan, China; 2 Department of Geriatrics, Tongji Hospital, Tongji Medical College, Huazhong University of Science and Technology, Wuhan, China

**Keywords:** adipokine, asprosin, adipose browning, adipogenesis, Nrf2

## Abstract

Obesity is an increasingly serious epidemic worldwide characterized by an increase in the number and size of adipocytes. Adipose tissue maintains the balance between lipid storage and energy utilization. Therefore, adipose metabolism is of great significance for the prevention, treatment and intervention of obesity. Asprosin, a novel adipokine, is a circulating hormone mainly secreted by white adipose tissue. Previous studies have shown that asprosin plays a role in fasting-induced homeostasis, insulin resistance, and glucose tolerance. However, whether it can regulate the metabolism of adipose tissue itself has not been studied. This study intended to examine the roles and potential mechanisms of asprosin in adipose regulation. We first demonstrated that the expression level of asprosin was significantly downregulated in subcutaneous white adipose tissue (scWAT) of high-fat diet (HFD)-fed or cold-stimulated mice. Overexpression of asprosin in scWAT reduced heat production, decreased expression of the browning marker uncoupling protein 1 (UCP1) and other browning-related genes, along with upregulation of adipogenic gene expression. Mechanistically, we found that Nrf2 was activated upon cold exposure, but this activation was suppressed after asprosin overexpression. In primary cultured adipocytes, adenovirusmediated asprosin overexpression inhibited adipose browning and aggravated lipid deposition, while Nrf2 agonist oltipraz could reverse these changes. Our findings suggest that novel adipokine asprosin negatively regulated browning and elevate lipid deposition in adipose tissue via a Nrf2-mediated mechanism. Asprosin may be a promising target for the prevention and treatment of obesity and other metabolic diseases.

## Introduction

Obesity and obesity-related disorders such as type 2 diabetes are steadily increasing worldwide with the improvement of people’s living standards and unhealthy lifestyle (Mohammed *et al.* 2018). Obesity is a pathophysiological condition that can lead to a variety of diseases, such as type 2 diabetes (Bhupathiraju & Hu 2016), cardiovascular diseases (Vecchie *et al.* 2018), non-alcoholic fatty liver disease (Polyzos *et al.* 2019) and even increased risk of cancers (Salaun *et al.* 2017). If energy intake exceeds energy expenditure, the energy imbalance will eventually lead to obesity (McQueen *et al.* 2018).

Tackling obesity requires reducing calorie intake first, followed by changes in metabolic efficiency (Camilleri & Acosta 2018), such as reducing fat synthesis and increasing energy expenditure in key metabolic organs, such as adipose tissue. Adipose tissue can be further divided into white (WAT) and brown (BAT) adipose tissue, which are distinct in form and function and have different metabolic characteristics (Bartelt & Heeren 2014). When exposed to certain environmental stimuli (such as cold exposure), adipocytes in white adipose tissue convert to a brownish phenotype, which is called white adipose tissue browning (Wang & Seale 2016). The browned white adipocytes begin to express uncoupling protein-1 (UCP1), a specific marker of brown adipose tissue, making white adipose tissue more likely to generate heat and increase the body’s energy consumption. In addition to the change of environmental temperature, many factors such as inflammation, oxidative stress and autophagy can affect the white adipose browning (Huerta-Delgado *et al.* 2020, Yamamuro *et al.* 2020). Browning of white adipose tissue is currently considered as a new approach to weight loss, insulin sensitivity and glucose tolerance, with potential therapeutic effects in the treatment of obesity and cardiovascular diseases.

It is well known that white adipose tissue is the main endocrine organ that secretes a variety of 'adipokines' including leptin, adiponectin, resistin, and asprosin (Fasshauer & Bluher 2015). Asprosin, the c-terminal propeptide of FBN1 (Muthu & Reinhardt 2020), is a novel adipokine secreted mainly by white adipose tissue (Romere *et al.* 2016) and plays an important regulatory role in the glucose metabolism of liver (Greenhill 2016), muscle (Jung *et al.* 2019) and pancreas (Lee *et al.* 2019, Wang & Hu 2020). Asprosin is also a centrally acting orexigenic hormone that increases appetite and ultimately leads to obesity and weight gain (Duerrschmid *et al.* 2017). However, its metabolic regulation on adipose tissue itself has not been studied.

Our findings indicated that the expression of asprosin was decreased during the browning process of white adipose tissue, while asprosin overexpression in white adipose tissue inhibited its browning, reduced the body’s thermogenesis, increased fat synthesis, and aggravated the lipid deposition in adipocytes by inhibiting the Nrf2 pathway. This physiological function of asprosin may make it a new therapeutic target for obesity and related diseases.

## Materials and methods

### Animals

All animal work was approved by the Institutional Animal Care and Use Committee of Huazhong University of Science and Technology. Fifty 10-week-old male C57BL/6J mice were randomly divided into two groups and administered adenoviruses (1 × 10^9^ plaque forming units) (Longchamp *et al.* 2018) expressing the entire coding sequence of murine asprosin (Ad-Asprosin) or a negative control virus encoding green fluorescent protein (Ad-GFP) in the groin subcutaneous white adipose tissue. Adenoviruses were injected into the center of inguinal scWAT pads within a marked area of approximately 0.5 cm in diameter. The injection site on each side was divided into 10 intensive sites to ensure that the adipose tissue in the labeled area could be overexpressed. After adenovirus injection, the mice were housed under 12 h light:12 h darkness cycle with* ad libitum* access to food and drinking water in a controlled temperature (23°C ± 1°C) for 2 days. Two days later, we exposed the mice to cold exposure (4°C). In the first 6 h, we measured their rectal temperature using a mouse thermometer at specified time points. After 24 h of acclimation (Huang *et al.* 2017), indicators related to respiratory metabolism were measured using Comprehensive Lab Animal Monitoring System (CLAMS) for the next 24 h. At day 5, the mice were sacrificed and tissues were collected to further examine the expression of asprosin and histomorphology. Adenovirus was recommended a period of 3–7 days following infection to assess effects on gene expression or physiology (Gomez-Banoy & Lo 2017).

HFD mice were fed with a diet containing 60% calories as fat (D12492, Research Diets, New Brunswick, NJ) for 12 weeks. Other mice used in this study were fed with the standard chow diet (D12450B, Research Diets, New Brunswick, NJ).

### Generation of recombinant adenovirus

Replication-defective recombinant adenovirus carrying the entire coding sequence of asprosin (Ad-Asprosin) with a signal peptide was constructed with the Adenovirus Expression Vector Kit (Takara Bio Inc., Kusatsu, Japan). Asprosin is a novel adipokine encoded by exon 65 and exon 66 of Fibrillin 1 (FBN1). In order to ensure its normal function and secretion, we constructed adenovirus encoding signal peptide (1-24aa) and propeptide (25-44aa), which can be found on UniProt (Q61554). An adenovirus-only-containing green fluorescence protein (GFP) was used as a negative control (Ad-GFP). Amplification and purification of recombinant adenovirus was designed and synthesized by OBiO Technology (Shanghai, China) Corp., Ltd.

### Metabolic analysis

Mice were individually kept in metabolic cages at 4°C under a 12 h light:12h darkness cycle, starting at 08:00 h. After 24 h of acclimation, the oxygen consumption rate (VO_2_), carbon dioxide production rate (VCO_2_), and physical activity were measured using Comprehensive Lab Animal Monitoring System (CLAMS) for the next 24 h, and each cage was monitored at a 15 min interval.

### Enzyme-linked immunosorbent assay

Enzyme-linked immunosorbent assay (ELISA) was performed to measure asprosin levels in mice serum by using commercial kits (Bioswamp, MU30851) according to the manufacturer’s instruction. Read the absorbance at 450 nm immediately after adding the stop solution. Quantitative results were calculated by standard curves.

### Western blot analysis

Cells or tissues were homogenized in ice-cold RIPA buffer with a proteinase inhibitor cocktail (Sigma-Aldrich). Protein concentrations were determined using the BCA Protein assay kit (Thermo Scientific). Equal amounts of protein were fractionated by 10% SDS polyacrylamide gels, followed by immunoblotting with the following primary antibodies: asprosin (FNab09797), UCP1 (ab10983; Abcam), PGC1a (ab54481; Abcam), β-tubulin (ab6046; Abcam). Membranes were then incubated with peroxidase-conjugated secondary antibody, and specific bands were detected with a Bio-Rad (Hercules, CA) imaging system.

### RNA extraction and qRT-PCR

Total RNA was extracted from cells or tissues with the use of TRIzol reagent (D9108A, Takara Bio). RNA was reverse-transcribed using the RNA PCR Kit (RR036A, Takara Bio). Quantitative PCR (qPCR) amplification was performed with an ABI PRISM 7900 Sequence Detector system (Applied Biosystem, Foster City, CA) according to the manufacturer’s instructions. Relative gene expression (normalized to 18S) was calculated using the comparative CT method formula 2^-∆∆CT^. The real-time PCR primer sequences are shown in Supplementary Table 1 (see section on supplementary materials given at the end of this article).

### Histological analysis

Histology experiments were performed exactly as described previously (Rao *et al.* 2019). Hematoxylin and eosin staining (H&E) of subcutaneous white adipose tissue samples were fixed in 4% paraformaldehyde overnight and embedded in paraffin. Paraffin blocks were sliced into 5 mm in thickness and stained with hematoxylin and eosin for 5 min. For IHC experiments, paraffin slides were incubated with 0.3% H2O2 for 10 min to quench the endogenous peroxidase, then incubated in 1% BSA (BSA) for 10 min, followed by incubation with UCP1-antibody at 4 C overnight. Afterwards, the slides were incubated with horseradish peroxidase (HRP)-conjugated secondary antibody at room temperature for 2 h. The images were photographed on a light microscope with image-pro-plus program.

### MEF culture, differentiation, and treatment

Primary Mouse embryonic fibroblasts (MEFs) were generated from pregnant mice at day 13.5 post coitum as described before (Braga *et al.* 2013). The embryonic head and internal organs were removed, the remaining carcasses were rinsed in 1× PBS and minced with scissors. Next, minced carcasses were suspended with 20ml 0.025% trypsin/EDTA (Roche) in a 50ml EP tube, and incubated in a water bath for an hour at 37°C. The tube was shaken every 15 min. The trypsin was neutralized by adding DMEM with 10% FBS, then passed through a 100μm nylon mesh cell strainer into a new tube to remove undigested tissues. Cell suspensions were centrifuged and resuspended in Dulbecco’s modified Eagle medium (DMEM) supplemented with 10% fetal bovine serum (FBS) and 1% penicillin and streptomycin. The cells were plated into a 10cm dish and cultured until confluent. Two days after confluence (day 0), the medium was changed to preadipocyte differentiation medium (DMEM supplemented with 10% FBS, 3-isobutyl-1-methylxanthine (0.5 mM, I5879, Sigma-Aldrich), insulin (10 μg/ml, 1342106, Sigma-Aldrich), dexamethasone (2 μM, D1756, Sigma-Aldrich), and rosiglitazone (2.5 μM, R2408, Sigma-Aldrich). From day 2, medium containing 10 μg/mL insulin and 2.5 μM rosiglitazone was changed every 2 days. The cells were divided into two groups and were infected with adenovirus on day 6 and the culture medium was changed to 2% (w/v) fatty acid-free BSA on day 8. Cells were treated with or without 8-Br-cAMP (1 mM, B5386, Sigma) for 4 and 12 h after 24 h pre-incubation with Oltipraz (10 μM, HY-12519, MCE). All cells were maintained in a 37°C and 5% CO2 humidified atmosphere.

### Red Oil O staining

Cell slides were stained with Oil Red O (O0625, Sigma) and counterstained with Mayer’s hematoxylin to visualize intracellular lipid droplets. All digital images were obtained with a light microscope (Olympus, Tokyo, Japan).

### Statistical analysis

Values are expressed as means of triplicate experiments ± s.e.m. Each series of experiments was repeated at least three times. Exclusion of individual data points was determined using an outlier calculator included in the Prism 7 software package (GraphPad Software Inc., CA) and excluded from analyses. Statistical analysis was performed using a Student’s t-test when two groups were compared or by one-way ANOVA, followed by Bonferroni* post hoc* tests, if multiple groups were compared. *P* value < 0.05 was considered statistically significant.

## Results

### Asprosin is mainly expressed in white adipose tissue and decreases in adipose metabolism

**Figure 1 fig1:**
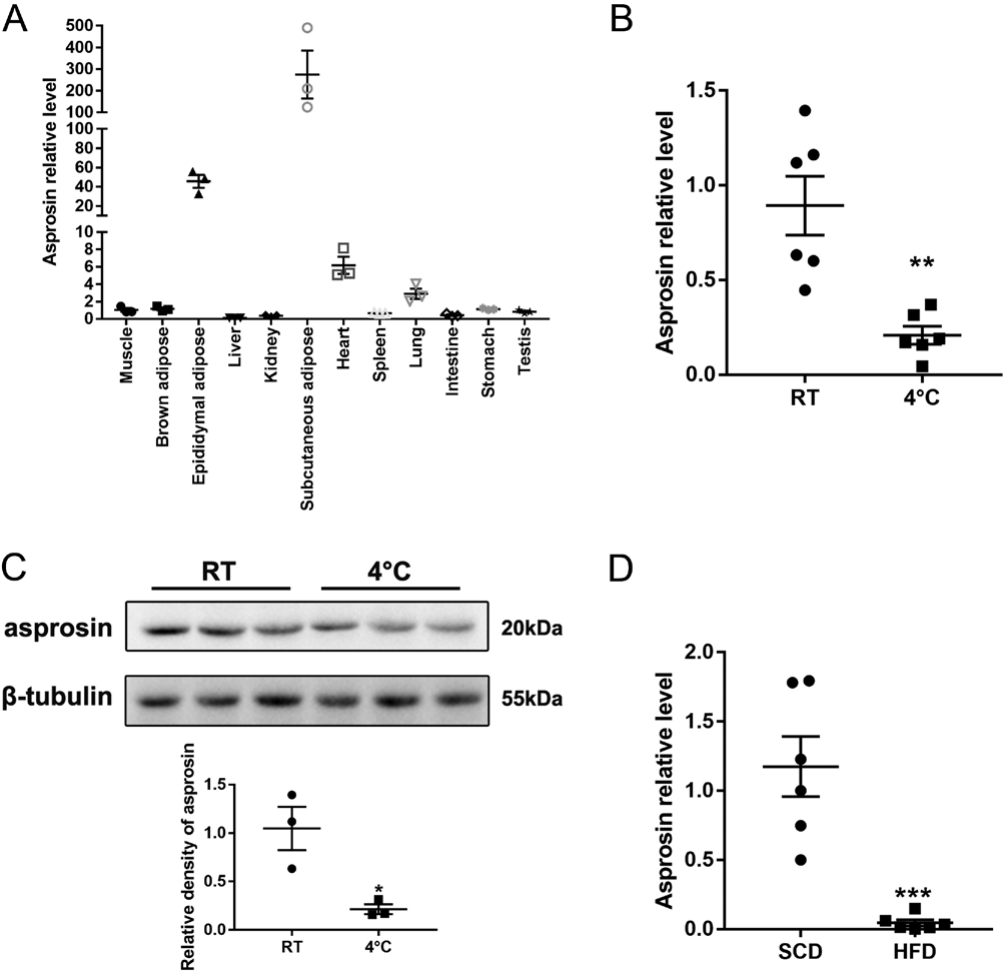
Decreased expression of asprosin in mouse white adipose tissue (WAT). (A) mRNA expression levels and distribution of asprosin in C57/BL6J mice (*n* = 3). The mRNA (B) and protein (C) expression of asprosin in the subcutaneous white adipose tissue (scWAT) of mice at room temperature (RT) or upon cold exposure (4°C) for 24 h (*n* = 3–6). (D) Asprosin mRNA level in scWAT of mice with standard chow diet (SCD) or high-fat diet (HFD) (*n* = 6). *P < 0.05, **P < 0.01 and ***P < 0.001 compared to the control group.

Asprosin was expressed predominantly in white adipose tissue (subcutaneous adipose and epididymal adipose), with a minor fraction in brown adipose tissue (BAT), liver, and muscle (Fig. 1A). To further explore whether asprosin was involved in adipose browning, we examined its expression levels in different models (cold exposure and high-fat diet, classic models of browning). First, we analyzed asprosin expression in subcutaneous white adipose tissue (scWAT) from mice maintained at room temperature (RT) or in a cold environment (4°C) and found that the expression of asprosin was decreased, at both mRNA and protein levels (Fig. 1B and C). Furthermore, as revealed in Fig. 1D,1in the subcutaneous tissue of mice fed with high-fat diet (HFD) for 12 weeks, the expression of asprosin was significantly decreased, compared to that of standard chow diet (SCD) control mice. These findings indicated that the novel adipokine asprosin may play an important role in adipose energy production and conversion.

### Asprosin overexpression decreases energy expenditure of mice during cold exposure

**Figure 2:**
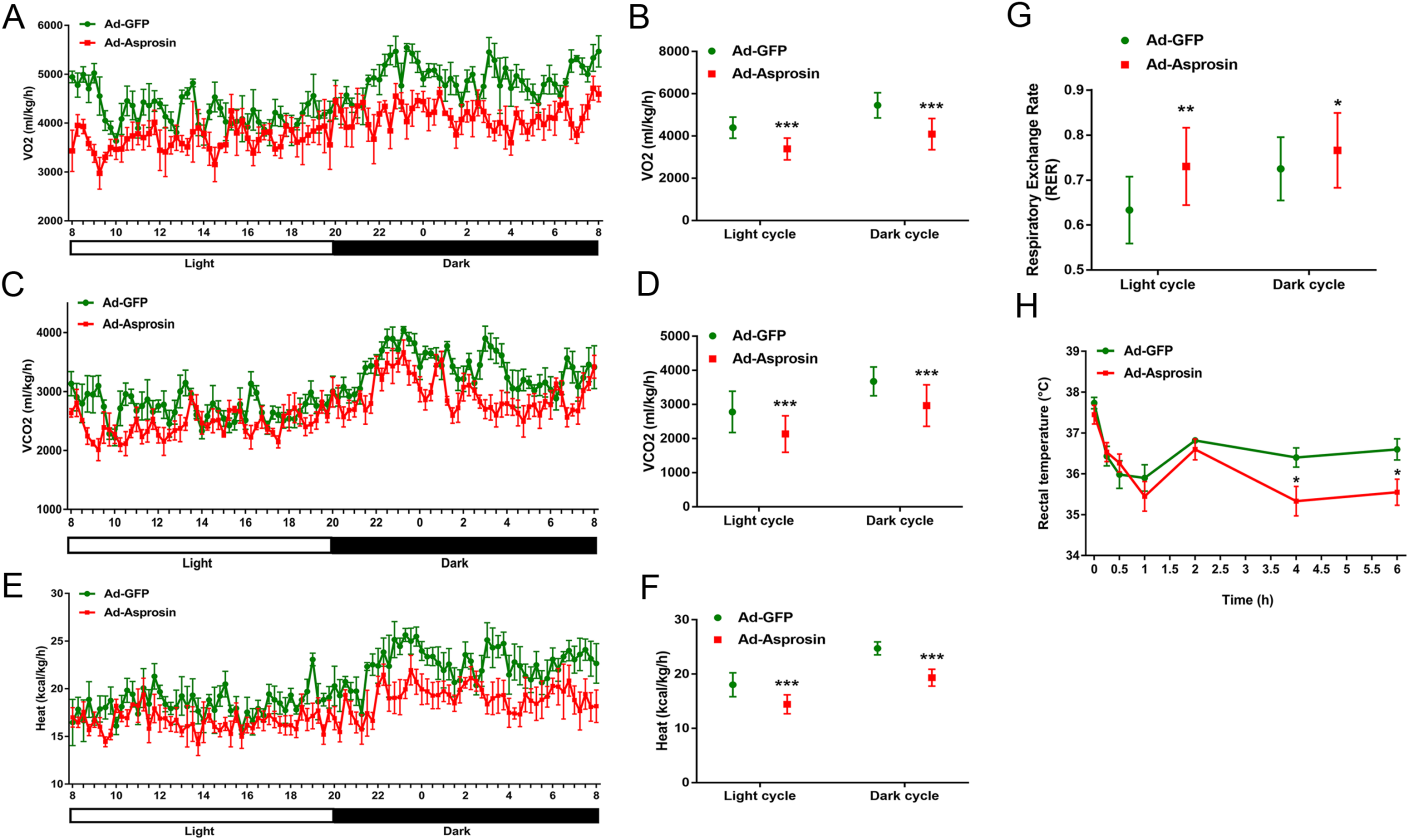
Asprosin overexpression decreases energy expenditure of mice during cold exposure. Mice injected with adenovirus overexpressing GFP (Ad-GFP) or asprosin (Ad-Asprosin) were housed individually at 4°C for a 12 h light:12 h darkness cycle. The whole-body oxygen consumption rate (VO_2_) (mL/kg/h) (A and B), carbon dioxide production (VCO_2_) (mL/kg/h) (C and D) and heat production (E and F) of mice measured by Columbus Oxymax metabolic chambers at 4°C and the average values were calculated by 12 h light:12 h darkness periods. (G) The average values of respiratory exchange ratio (RER) in the mice for a 24h cycle were calculated from the metabolic cage data. (H) Rectal temperature of the mice was recorded at the indicated time points upon cold exposure (4°C) (*n* = 6–8). *P < 0.05, **P < 0.01 and ***P < 0.001 compared to the control group. A full color version of this figure is available at https://doi.org/10.1530/JOE-20-0503.

**Figure 3:**
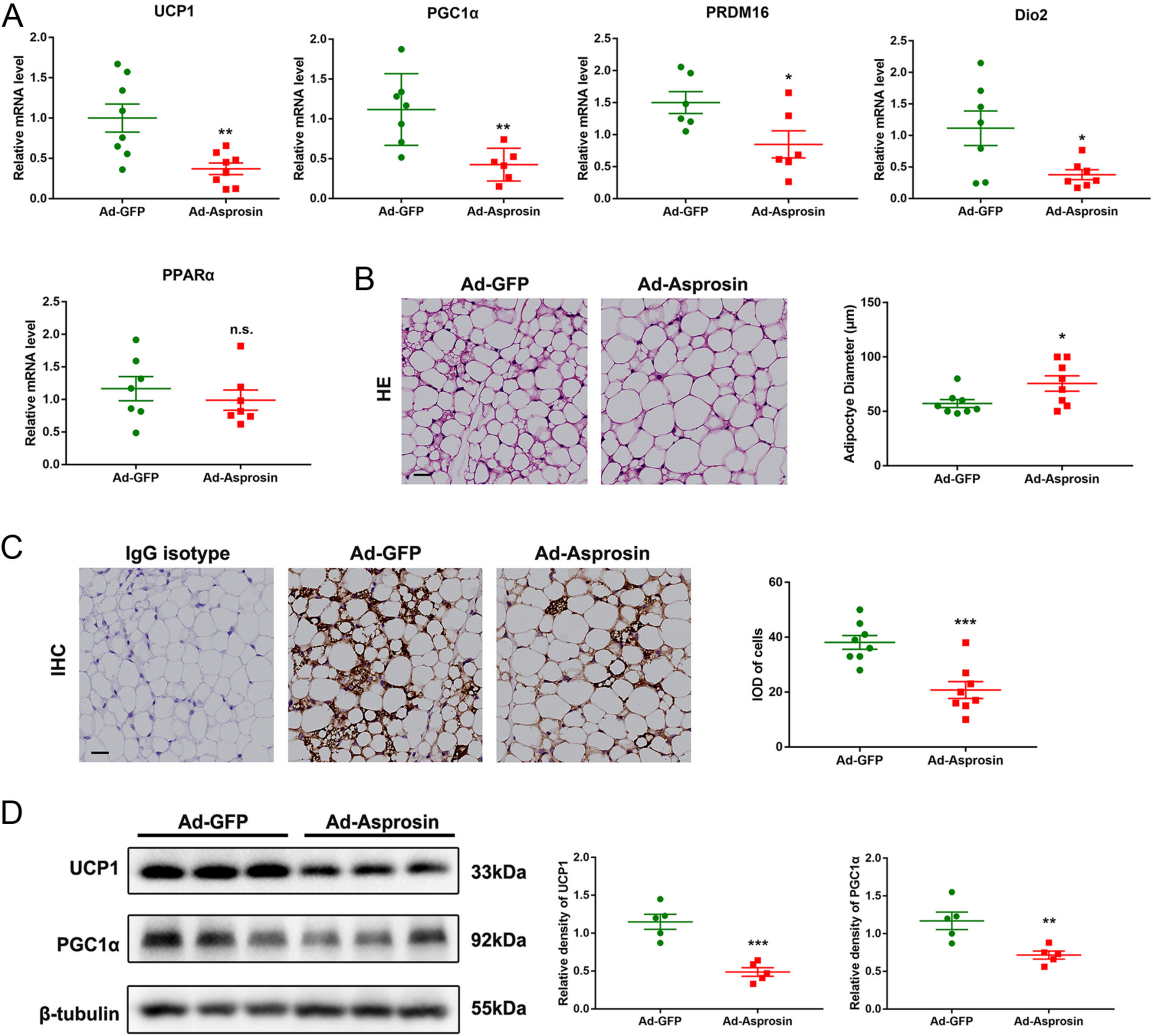
Asprosin overexpression inhibits the development of browning in white adipose tissue. Mice were treated as indicated in Fig. 2. (A) Effects of asprosin overexpression on the expression of adipose browning factors (UCP1, PGC1a, PRDM16, Dio_2_ and PPARa) in the scWAT of cold-treated mice, as determined by qRT-PCR (*n* = 6–8). (B) H&E staining of scWAT of Ad-GFP or Ad-Asprosin mice and adipocyte size distribution. Quantification of adipocyte size was performed by ImageJ software (*n* =8). (C) Immunohistochemistry analysis of UCP1 staining (brown stain) in scWAT sections (*n* = 8). (D) Western blot analysis for UCP1 and PGC1a in the scWAT of experimental mice (*n* = 5). *P < 0.05, **P < 0.01 and ***P < 0.001 compared to the control group. n.s. means no statistical significance. A full color version of this figure is available at https://doi.org/10.1530/JOE-20-0503.

We next determined whether asprosin overexpression affected energy balance. Recombinant adenovirus (Ad-Asprosin) were generated and locally injected into the groin subcutaneous white adipose tissue to elevate the level of asprosin* in vivo* (Supplementary Fig. 1B, C and D). The whole-body oxygen consumption rate (VO_2_) (Fig. 2A and B) and carbon dioxide production rate (VCO_2_) (Fig. 2C and D) decreased in Ad-Asprosin mice compared to those in Ad-GFP control mice when mice were exposed to cold. Whole-body energy expenditure, which was represented by heat production, was significantly blunted in Ad-Asprosin mice (Fig. 3E and F). The basal respiration exchange ratio (RER), indicating the relative proportion of carbohydrates vs lipids as a substrate for energy consumption (18), by calculating VCO_2_/VO_2_, were increased in Ad-Asprosin mice (Fig. 2G), meaning a lower availability of fatty acids. Additionally, by measuring the rectal temperature of mice, we detected that asprosin overexpression in mice adipose tissue made them more difficult in maintaining their body temperature. During cold exposure, the core temperature of Ad-GFP control mice displayed better body temperature conservation (37.73 ± 0.28°C to 36.4 ± 0.74°C), while the Ad-asprosin mice dropped rapidly from 37.45 ± 0.38°C to 35.55 ± 1.05°C in the first 6h (Fig. 2H), suggesting that overexpression of asprosin cut down energy expenditure via reduced thermogenesis.

### Asprosin overexpression inhibits the development of browning in white adipose tissue

When stimulated by cold, white adipocytes convert to beige adipocytes. The main feature of beige adipocyte is that they produce heat, keeping the mice’s body temperature stable during cold stimuli.

In order to further explore the relationship between the difficulty in maintaining body temperature and the overexpression of asprosin in mice, we analyzed the gene expression of classical browning markers including UCP1, PGC1a, PRDM16, Dio_2_ and PPARa, and the results revealed that most of the markers were significantly decreased in the scWAT of the asprosin overexpression group (Fig. 3A). Meanwhile, asprosin overexpression in WAT led to an decreased adipocytes size in scWAT (Fig. 3B). IHC analysis of UCP1 staining (brown stain) of scWAT sections also showed less UCP1+ cells in Ad-Asprosin mice (Fig. 3C). Consistent with the above results, Western blot analysis of UCP1 and PGC1a, typical markers of thermogenesis, showed decreased protein levels in scWAT from Ad-Asprosin mice compared to those in Ad-GFP control mice after exposure to cold (Fig. 3D). These data demonstrated that asprosin significantly suppressed being in WAT.

### Nrf2 participates in asprosin mediated inhibition of adipose browning

**Figure 4:**
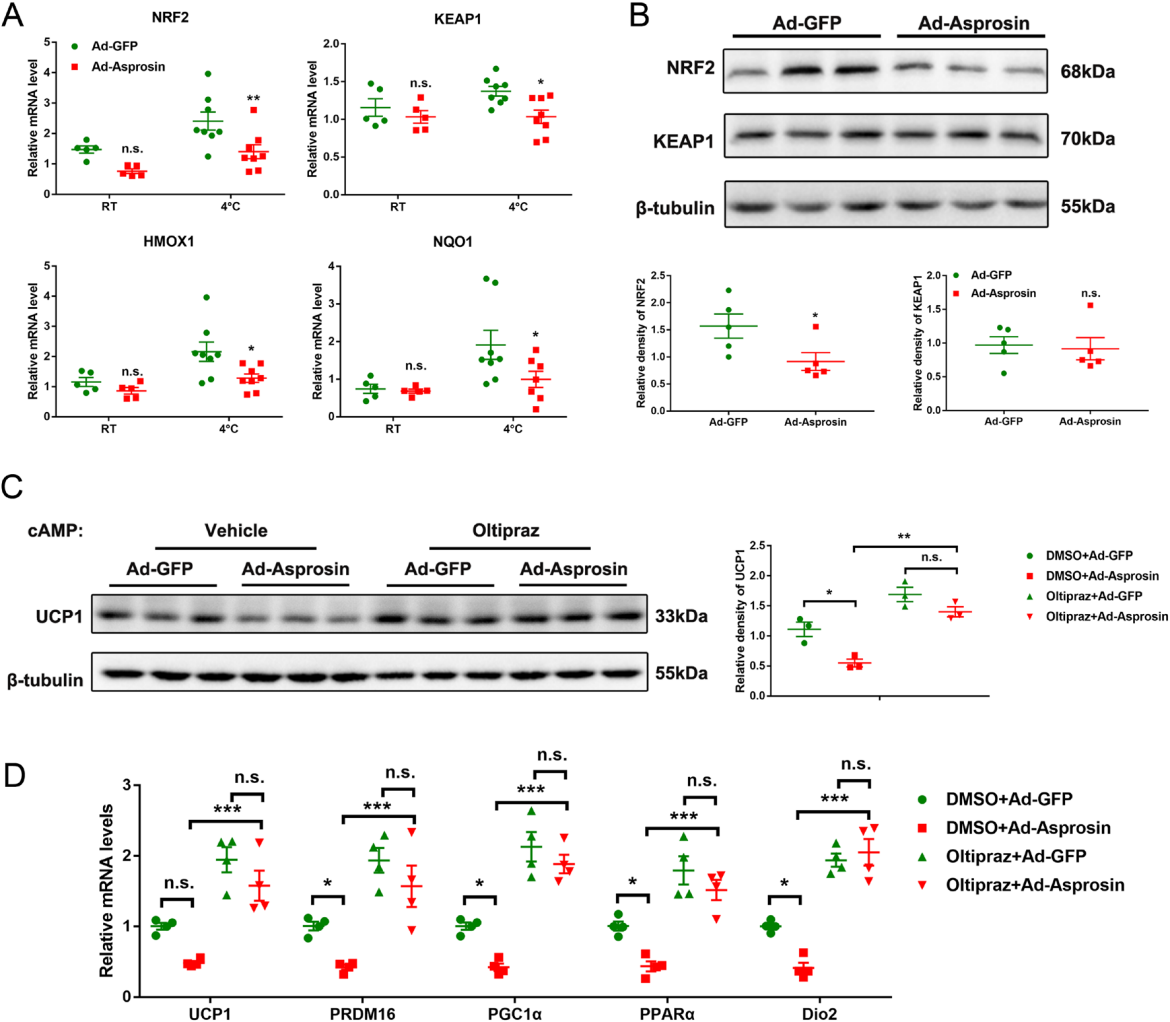
Nrf2 participates in asprosin mediated inhibition of adipose browning. (A) Relative mRNA levels of the Nrf2, Keap1 and Nrf2 targets Hmox1 and Nqo1 in mice scWAT. (B) Western blot analysis of Nrf2 and Keap1 in scWAT from 4°C treated mice (*n* = 5–8). (C and D) After adenovirus transfection, adipocytes were preincubated with oltipraz 10 µM for 24 h and then stimulated with 8-Br-cAMP 1 mM for 4 h. Oltipraz treatment elevated Nrf2 activity and rescued the inhibition of asprosin overexpression on thermogenesis in response to 8-Br-cAMP in adipocytes (*n* = 3–4). (C and D) The temperature condition was RT. (C) Protein levels of UCP1 in adipocytes were detected by Western blot. (D) Relative mRNA levels of browning related genes in cultured adipocytes with different treatments. *P < 0.05, **P < 0.01 and ***P < 0.001 compared to the control group. n.s. means no statistical significance. A full color version of this figure is available at https://doi.org/10.1530/JOE-20-0503.

To uncover the underlying mechanism of the aforementioned observations, we explored whether Nrf2, a crucial antioxidant transcription factor, was involved in the process. Nrf2 is a transcription factor known for its antioxidant properties, with the development of research, it has been found that Nrf2 plays a pivotal role in adipose biology, both directly and indirectly (Schneider & Chan 2013). The activation of Nrf2 is coordinated by specific repressor Kelch-like ECH-associated protein 1 (Keap1) (Wang *et al.* 2020). We measured the expression levels of Nrf2 and Keap1, both the mRNA and protein levels of Nrf2 were downregulated in Ad-Asprosin mice, together with its downstream target genes, however, the change of Keap1 expression level was not significant (Fig. 4A and B). To further verify the involvement of Nrf2 signaling in the cellular-autonomous regulation of asprosin on thermogenesis, cAMP and oltipraz (an Nrf2 activator) (Zhao *et al.* 2018) was applied to the subsequent cell experiments, cAMP is a ubiquitous second messenger that plays an important role in a wide range of cells and multiple signaling pathways. The cAMP signaling pathway is very important for early brown adipocytes differentiation by promoting the proliferation and expression of transcription factors, including key thermogenic regulators (Reverte-Salisa *et al.* 2019). As shown in Fig. 4C, Western blot showed that the protein levels of UCP1 were lowered by Ad-Asprosin when compared to that of the Ad-GFP control, while oltipraz could rescue the inhibition of asprosin overexpression on thermogenesis in adipocytes. In parallel, we also tested the effects of oltipraz on the expression of browning markers including UCP1, PGC1a, PRDM16, Dio_2_ and PPARa and got a consistent conclusion (Fig. 4D). These observations suggested that asprosin refrain adipose browning through an Nrf2-mediated mechanism.

### Asprosin promotes adipogenesis in primary adipocytes in vitro through Nrf2 pathway

**Figure 5 fig5:**
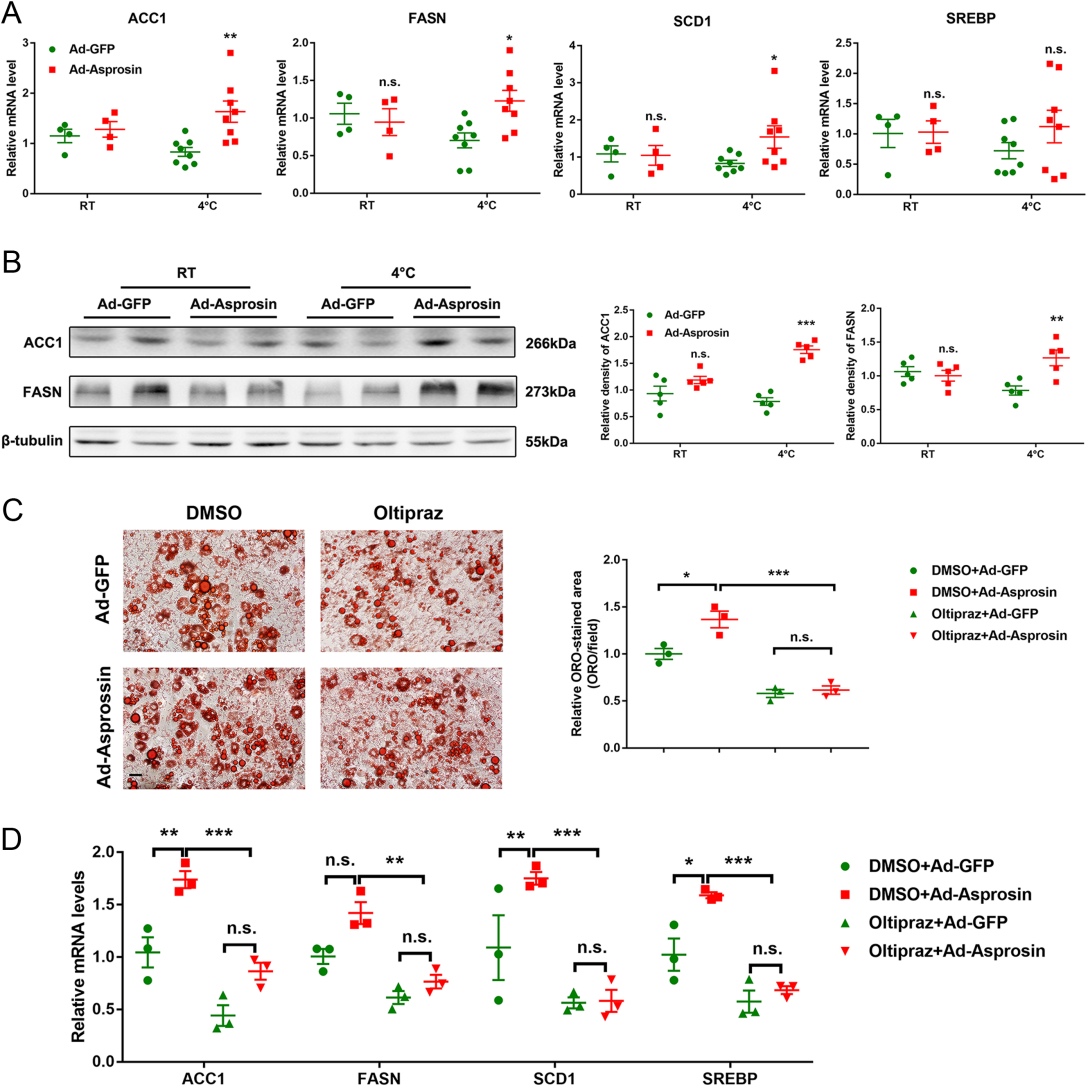
Asprosin promotes adipogenesis in primary adipocytes* in vitro* through Nrf2 pathway. (A) Relative mRNA expression of lipogenesis-related genes in mice scWAT (*n* = 4–8). (B and C) Lipogenesis was rescued by oltipraz treatment in Ad-Asprosin cells (*n* = 3). (B) Western blot analysis of ACC1 and FASN in the scWAT of experimental mice (*n* = 5). (C) Representative images of ORO staining in adipocytes treated with Ad-GFP or Ad-Asprosin, with or without oltipraz treatment, was performed on day 8 after induction of differentiation. Scale bar is 50 μm. Quantification of relative ORO-stained area is shown in the graphs at right. (D) Lipogenesis-related genes were analyzed by RT-qPCR. The temperature condition was RT. *P < 0.05, **P < 0.01 and ***P < 0.001 compared to the control group. n.s. means no statistical significance. A full color version of this figure is available at https://doi.org/10.1530/JOE-20-0503.

Nrf2 also plays an important role in adipogenesis (Haider & Larose 2020). In previous studies, we observed that overexpression of asprosin could reduce the expression of Nrf2, inhibit its activity and downregulate its downstream target genes. Therefore, we further examined the mRNA levels of adipogenesis gene expression, including ACC1, FASN, SCD1 and SREBP, as displayed in Fig. 5A,5almost all these genes were elevated in Ad-Asprosin mice when upon cold exposure except for SREBP. Consistent with the increased mRNA levels, the protein expression of ACC1 and FASN were also upregulated by Ad-Asprosin (Fig. 5B), along with higher free fatty acid (FFA) release in mice serum (Supplementary Fig. 1F). Red Oil O staining of adipocyte also showed that overexpression of asprosin increases lipid synthesis and reduces lipolysis in adipocytes, which can be reversed by Nrf2 agonists oltipraz (Fig. 5C). Accordingly, the regulation of overexpression of asprosin on lipid synthesis in adipocytes with or without oltipraz treatment was also determined. As shown in Fig. 5D,5the adipogenic gene expressions were upregulated in adipocytes with asprosin overexpression, but the trend was reversed with Nrf2 activation. Altogether, these findings indicate that asprosin not only inhibits browning and thermogenesis of adipose tissue, but also can aggravate lipid deposition in adipocytes.

## Discussion

White adipocytes play a key role in maintaining whole-body energy homeostasis by forming white adipose tissue (WAT) (Schneider & Chan 2013). Browning of WAT has been recognized as an effective strategy for the treatment of obesity or adiposity (Li *et al.* 2017). Adipose tissue, as an endocrine organ, can secrete a variety of metabolic regulators, among which adipokines such as adiponectin and leptin have been proved to be related to the browning of white adipose tissue (Dodd *et al.* 2015, Hui *et al.* 2015). Asprosin is such a newly discovered adipokine thatin is mainly secreted by white adipose tissue (Dodd *et al.* 2015). Studies on asprosin mainly focused on glucose homeostasis (Li *et al.* 2019), it has been reported that asprosin is related to a range of metabolic diseases such as non-alcoholic fatty liver disease (Ke *et al.* 2020), insulin resistance (Yuan *et al.* 2020), but its role in adipose tissue remains unknown.

In this study, we found that the expression level of asprosin was significantly downregulated in subcutaneous white adipose tissue of HFD-fed or cold-stimulated mice, which seems to be contradicted from several other results reporting elevated 'plasma' asprosin in obese mice, and humans (Duerrschmid *et al.* 2017). However, when asprosin was overexpressed in the scWAT, the cold-stimulated mice displayed attenuated metabolic phenotype (decreased energy expenditure, O2 consumption and CO2 production rate, concomitant with reduced heat production). These phenomenons indicate that asprosin negatively regulates the browning of white adipose tissue. We suggest that asprosin mRNA levels may be reduced through negative feedback. Consistent with previous studies, we also examined the expression of asprosin and found that the expression of asprosin was increased in adipose tissue of obese db/db mice (Supplementary Fig. 1A), which may be the result of negative feedback regulation blocking due to the 'leptin receptor' gene knockout. This finding provided the first evidence that novel adipokine asprosin in adipose tissue may participate in the pathophysiological and physiological processes of obesity.

Mechanistically, overexpression of asprosin suppressed Nrf2 activity in the adipose tissue, which may partially explain the underlying mechanism for asprosin on adipose function. Nuclear factor Erythroid 2-related factor 2 (Nrf2), a member of the NC-BZIP protein family, collaborates with the small muscle fascia fibrosarcoma protein to transcriptional control genes containing antioxidant reaction elements (ARE) (Wang *et al.* 2020). Nrf2 activation is coordinated by a specific inhibitory factor kelch-like ech-related protein 1 (Keap1), which regulates gene networks and controls a variety of homeostasis processes including adaptive antioxidant reaction and detoxification (Bellezza *et al.* 2018). Interestingly, a growing body of evidence suggests that Nrf2 may regulate adipose tissue formation and function as a transcription factor, including adipocyte function, lipid metabolism and insulin sensitivity (Wang *et al.* 2020).

Contrary to our expectations, Nrf2 activity was significantly decreased, while Keap1 expression level showed no significant difference. We speculated that asprosin may directly affect the stability of Nrf2 protein or the transcriptional regulation of its downstream target genes, instead of indirectly affecting Nrf2 expression by adjusting Keap1. Further works need to be done to confirm our guesses.

Nrf2 also exhibits profound effects on adipogenesis. Suppression of Nrf2 activity, genetically or chemically, leads to impaired adipogenesis in preadipocytes (Pi *et al.* 2010, Chen *et al.* 2013). Consistent with previous studies, mRNA levels of pivotal adipogenic gene expression in scWAT were also upregulated in Ad-Asprosin mice especially upon cold exposure. The increased lipid droplet was further supported by Red Oil O staining. Whereas the Nrf2 agonist oltipraz was administered while overexpressing asprosin, the elevation of lipid deposition in cultured adipocytes was suppressed.

**Figure 6:**
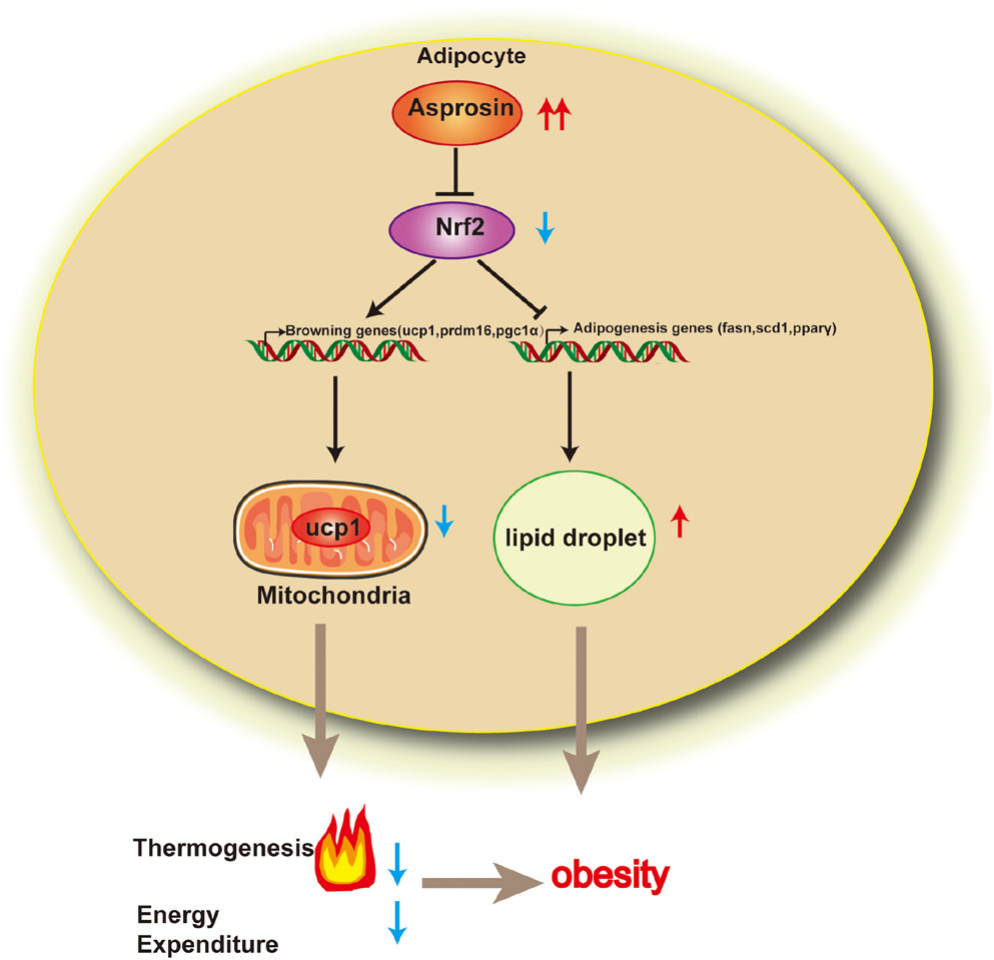
Asprosin modulates browning and adipogenesis in white adipose tissue through Nrf2-mediated mechanism. Asprosin expression was downregulated during white adipose browning. However, overexpression of asprosin in white adipose tissue inhibited its browning, reduced the body’s thermogenesis, increased fat synthesis, and aggravated the lipid deposition in adipocytes by inhibiting the Nrf2 pathway. A full color version of this figure is available at https://doi.org/10.1530/JOE-20-0503.

Taken together, mice with asprosin overexpression in scWAT inhibited adipose browning and aggravated lipid deposition* in vitro* and* in vivo*, along with the inhibition of Nrf2 activity (Fig. 6). Nrf2 agonist oltipraz could reverse these changes. However, the underlying mechanism needs to be further studied. Another limitation of our study is that we did not have an* in vivo* anti-asprosin antibody treatment, as adverse reactions, including hypersensitivity reactions and renal function impairment, caused by antibody treatment* in vivo* have been reported in several articles (Bavbek & Lee 2017, Guo *et al.* 2018). If a better method can be found in the future, we will further verify the metabolic benefits of asprosin knockdown* in vivo*.

Our study demonstrated that novel adipokine asprosin is a physiological regulator of adaptive thermogenesis. Overexpression of asprosin in scWAT inhibited browning and energy consumption and increased lipid deposition in adipose tissue, which makes asprosin a potential therapeutic target for obesity and other metabolic disorders.

## Supplementary Material

Figure S1. (A) The mRNA level of asprosin in the subcutaneous white adipose tissue (scWAT) of db/db mice compared with WT (wild type) (n=7). (B)Asprosin expression level in mice serum (n=10). (C and D) Recombinant adenovirus (Ad-Asprosin) significantly increased the protein and mRNA level of asprosin in subcutaneous white adipose tissue (scWAT) (n=8). (E) Expression level of asprosin mRNA was significantly higher in adipose tissue overexpressed by local injection than in uninjected sites (n=9). (F) Free fatty acid (FFA) release in mice serum were tested (n=6). (G) Effective activation of Nrf2 after oltipraz stimulation measured by western blot analysis (n=6). *P˂0.05, **P˂0.01 and ***P˂0.001 compared to the control group. “n.s.” means no statistical significance.

Table 1 Real-time primer sequences for genes of interest.

## Declaration of interest

The authors declare that there is no conflict of interest that could be perceived as prejudicing the impartiality of the research reported.

## Funding

This study was supported by the National Natural Science Foundation of China (81830014, 82000424).

## Author contribution statement

The authors have made the following declarations about their contributions: Caijun Rao and Yanli Miao conceived and designed the experiments. Yanli Miao, Haojie Qin, and Yi Zhong performed the experiments. Kai Huang and Caijun Rao analyzed the data and contributed reagents/materials/analysis tools. Yanli Miao and Caijun Rao drafted and revised the manuscript. Yanli Miao and Haojie Qin contributed equally to this work.
