# Supplementary figures and images for: Novel adipokine asprosin modulates browning and adipogenesis in white adipose tissue
Source: J Endocrinol. 2021 Mar 9;249(2):83–93. doi: 10.1530/JOE-20-0503 (PMC8052515; doi:10.1530/JOE-20-0503)

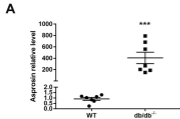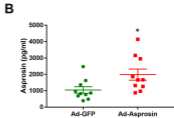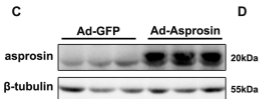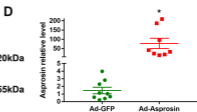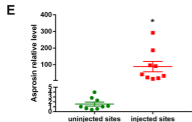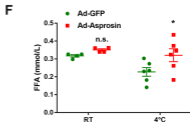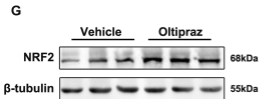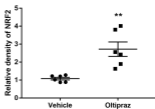

Supplement: Figure S1. (A) The mRNA level of asprosin in the subcutaneous white adipose tissue (scWAT) of db/db mice compared with WT (wild type) (n=7). (B)Asprosin expression level in mice serum (n=10). (C and D) Recombinant adenovirus (Ad-Asprosin) significantly increased the protein and mRNA level of asprosi [file supplementary_figure_1.pdf]
