# Supplementary material for: Novel adipokine asprosin modulates browning and adipogenesis in white adipose tissue
Source: J Endocrinol. 2021 Mar 9;249(2):83–93. doi: 10.1530/JOE-20-0503 (PMC8052515; doi:10.1530/JOE-20-0503)
Supplement: Table 1 Real-time primer sequences for genes of interest. [file supplementary_table_1.pdf]

Table 1 Real-time primer sequences for genes of interest.

**Primer name**	**Sequence** (5' to 3')
Asprosin forward	CAAGAGACGGAGAAGCACGA
Asprosin reverse	GCAGGAGCTCTAGGATTCGG
Ucp1 forward	GTGAACCCGACAACTTCCGAA
Ucp1 reverse	TGCCAGGCAAGCTGAAACTC
Pgc1α1 forward	CACTTCAATCCACCCAGAAAGCT
Pgc1α1 reverse	GGACATGTGCAGCCAAGACTCT
Prdm16 forward	CAGCACGGTGAAGCCATTC
Prdm16 reverse	GCGTGCATCCGCTTGTG
Dio2 forward	CAGTGTGGTGCACGTCTCCAATC
Dio2 reverse	TGAACCAAAGTTGACCACCAG
Fasn forward	GGTCGTTTCTCCATTAAATTCTCAT
Fasn reverse	CTAGAAACTTTCCCAGAAATCTTCC
Srebp1 forward	TGACCCGGCTATTCCGTGA
Srebp1 reverse	CTGGGCTGAGCAATACAGTTC
Scd1 forward	TCGCCCCTACGACAAGAACA
Scd1 reverse	CCGGTCGTAAGCCAGGCCCA
Pparγ forward	TCGCTGATGCACTGCCTATG
Pparγ reverse	GAGAGGTCCACAGAGCTGATT
Fabp4 forward	AAGGTGAAGAGCATCATAACCCT
Fabp4 reverse	TCACGCCTTTCATAACACATTCC
Pparα forward	CAGGAGAGCAGGGATTTGCA
Pparα reverse	CCTACGCTCAGCCCTCTTCAT
Cebpα forward	CCCAGCGGTGCCTTGTGC
Cebpα reverse	TCCTTCCCCCAGCCGTTAGTG
Nrf2 forward	TCTTGGAGTAAGTCGAGAAGTGT
Nrf2 reverse	GTTGAAACTGAGCGAAAAAGGC
Keap1 forward	TGCCCCTGTGGTCAAAGTG
Keap1 reverse	GGTTCGGTTACCGTCCTGC
18S forward	TTGACGGAAGGGCACCACCAG
18S reverse	GCACCACCACCCACGGAATCG
Acc1 forward	GATGAACCATCTCCGTTGGC
Acc1 reverse	GACCCAATTATGAATCGGGAGTG
